# Recurrent neural network long short term memory model to detect the pile toe using raw data of pile integrity test

**DOI:** 10.1038/s41598-026-36732-7

**Published:** 2026-02-12

**Authors:** Reham M. Samaan, Mohamed S. A. Saafan, Abdelsalam A. Mokhtar, Ahmed M. Ebid

**Affiliations:** 1 Faculty of Engineering, Ain Shams University, Cairo, Egypt; 2 Faculty of Engineering and Technology, Future University in Egypt, New Cairo, Egypt

**Keywords:** Pile integrity testing, Recurrent neural network, Long short-term memory, Reflectogram, Engineering, Mathematics and computing, Neuroscience

## Abstract

This article proposes a novel approach to automatically generate velocity reflectogram of Pile Integrity Testing using a Recurrent Neural Network with Long Short-Term Memory (RNN-LSTM) model. Conventional Low-Strain Integrity Testing (LSIT) accuracy relies significantly on expert interpretation of reflected wave signals and entails subjectivity as well as efficiency limitations. The purpose of this study is to develop an artificial intelligence system capable of learning wave propagation behavior from acceleration inputs and generating reflectogram that capture pile toe locations correctly, thereby reducing dependence on human experience. The proposed technique eliminates human error and increases both the reliability and efficiency of the model. The strategy involved the collection of LSIT data from several of Egypt’s driven piles projects, followed by systematic preprocessing which converted raw acceleration signals into digitized velocity–time series. Several RNN-LSTM networks with various hidden layers and neurons were trained and optimized against performance including measures the coefficient of determination (R^2^), computational expense, and visual examination of reflectogram. The proposed six-layer, 32-neuron LSTM model achieved an optimum balance between accuracy and computational expense and yielded training and validation R^2^ of 0.9126 and 0.8778, respectively, and demonstrated satisfactory predictive generalization. Visual examinations also guaranteed the validity of the model, where “Good” predictions for toe location were up to 84% for the validation set and 89.5% for the training set, while “Fair” and “Bad” predictions had an average of only 10% and 5%, respectively. The experiments demonstrate that the RNN-LSTM model effectively mimics human-generated reflectogram with high accuracy and low mis-adoption risk. Lastly, this research describes how deep learning, namely RNN-LSTM, presents an excellent alternative to the conventional generated reflectogram, greater reliability, and reduced reliance on human experience.

## Introduction

### Background

#### Pile integrity test

**Fig. 1:**
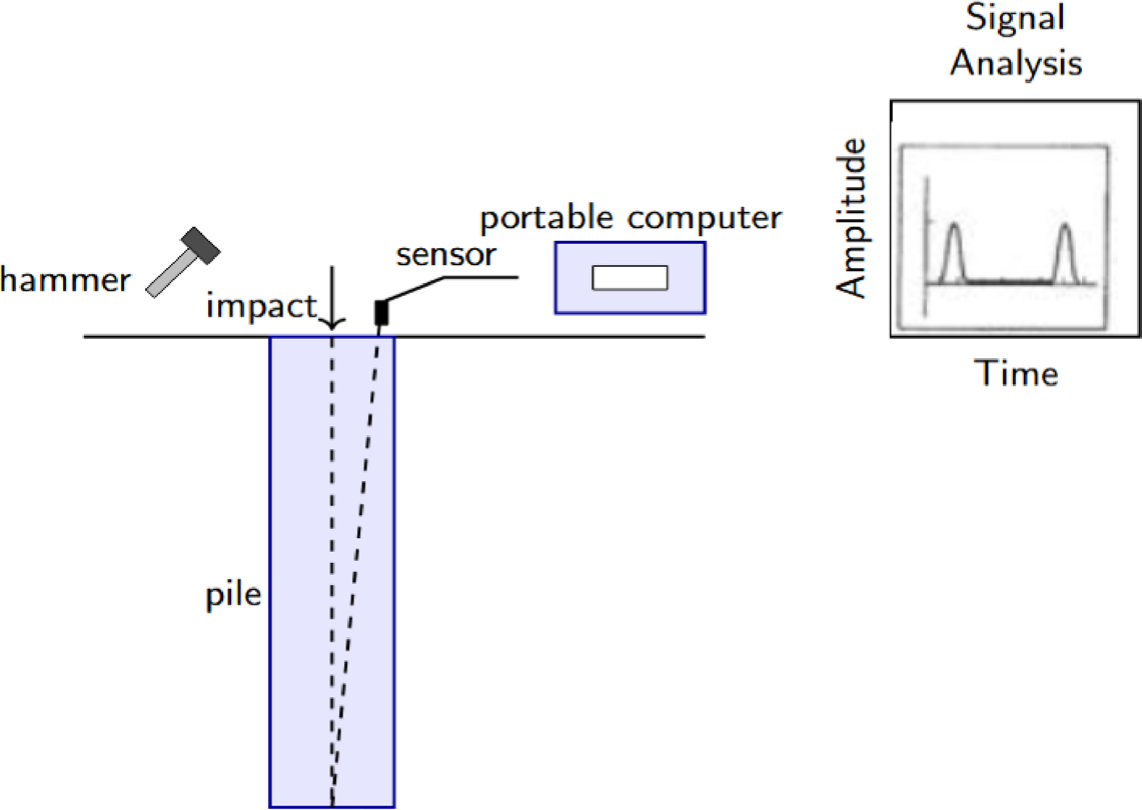
Schematic diagram of apparatus for pile integrity testing.

Pile Integrity Testing (PIT) or Low-Strain Integrity Testing (LSIT) is a non-destructive testing (NDT) used to measure the quality, structural integrity, continuity, and approximate length of concrete piles^1^^,^^2^. Some of the common methods include sonic echo (impact-echo), cross-hole sonic logging, and the sine-sweep excitation method, which confirms the length, continuity, and quality of the pile by testing the behavior in which waves travel and are reflected. The data are analyzed to identify defects and ensure the integrity of the piles^3^. LSIT with a hammer is a conventional technique for verifying the quality of various pile types^4^. The LSIT usually induces low-energy transient vibration, producing low-amplitude oscillations in the elastic range of pile material. The vibrations, produced without causing damage, allow pile integrity to be evaluated by inspecting reflected wave signals through testing. The signals are analyzed by using velocity or acceleration sensors and examined in the framework of elastic wave theory^5^. The sensor and motion signals are sent into a device that records, analyzes, and displays the data. The hammer strike produces stress waves that propagate down the pile and are reflected to the sensor, as indicated in Fig. 1.

The apparatus captures signals, eliminates noise, and amplifies the response of motion with time to facilitate interpretation. In the signal analysis, a signal output is displayed, showing the wave reflections within the pile. PIT utilizing low-strain methods adheres to standards that define procedures for testing, data interpretation, and reporting, ensuring the accurate assessment of pile condition. Standards like ASTM D5882^6^ and BS EN ISO 22,477–4:2018, which follow EN 1997–1 (Eurocode 7)^7^, both of which outline the steps for assessing integrity pile by using wave reflection analysis and standardized data processing.

The PIT depends mainly on the one-dimensional theory of stress waves, which explains the propagation of axial stress waves along the pile due to a low-strain impact. When it strikes the pile at the pile top, a compressive wave will travel downwards along the pile with speed given by the equation, which is Vp = E/ρ, where E is Young’s modulus and ρ is the material density. The second-order partial differential equation describes this wave propagation, as shown in Eq. (1).

1 $$\partial^{2} u/\partial t^{2} = c^{2} \left( {\partial^{2} u/\partial x^{2} + \partial^{2} u/\partial y^{2} + \partial^{2} u/\partial z^{2} } \right)$$

where u represents the wave’s amplitude, t is time, x, y, and z represent spatial coordinates, and c is the speed of wave propagation^8^. The force from the impact is directly related to the velocity v through the pile impedance, expressed as F = Z. v , where the pile impedance Z is calculated from Eq. (2), which shows that the movement in the pile depends on the relationship between the hammer’s impedance and the pile’s impedance^9^.

2 $$Z = \rho \cdot A \cdot V\_p$$

where A represents the cross-sectional area of the pile.

ASTM D5882 -96 defines the PIT as a method to evaluate single-pile integrity through low-strain impact procedures that measure velocity. The pile top must receive proper preparation before testing by ensuring it remains clean and intact while sensors are securely attached with bonding agents. The testing process should occur at several locations on top of the pile for piles over 500 mm in diameter. The testing is performed no earlier than 7 days following casting or until the concrete has achieved approximately 75% of its strength. The testing method involved an axial impact near the motion sensor using a handheld hammer, and the test results were provided using the accelerometers. Signals are delivered, and multiple impacts are analyzed to eliminate any noise to improve data reliability. The data collected is affected by several factors, including changes in cross-sectional, pile geometry, and material variability. For the determination of pile toe response, the data analysis is generally processed includes reflectogram records, estimated pile lengths, assumed wave velocities, and amplification functions. Regarding the assessment of the integrity of the structure, expert engineers determine impedance changes by examining signal reflections and comparing the data collected on the same piles. Also, any detected defects can be accurately measured computationally according to the data collected. The final interpretation depends on the engineer’s experience, who would then consider the mathematics along with relevant information, including testing records, soil condition, and loading requirements^5^. The mechanical characteristics of the pile and the surrounding soil significantly influence the propagation and reflection of stress waves in LSIT. Variations in soil stiffness, particle shape, and contact behavior can affect the impedance contrast and the intensity of reflected signals. Yuan et al.^10^ demonstrated that particle size distribution and treatment affect the mechanical behavior of engineering muck-based geopolymers. Yuan et al.^11^ examined the cyclic behavior of calcareous sand and showed deformation sensitivity associated with particle crushing during repeated loading. Yuan et al.^12^ studied pile-soil interaction under lateral loading in coral sand. They focused on the influence of granular composition on stress transfer and stiffness deterioration. These investigations show that the physical properties of geomaterials influence stress-wave behavior.

#### Recurrent neural network

**Fig. 2:**
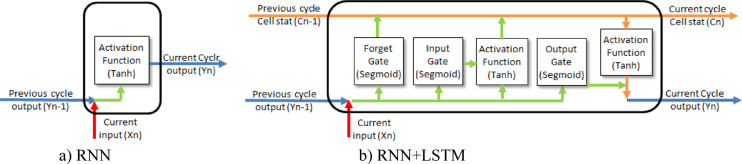
Schematic diagram of RNN and RNN + LSTM.

RNNs are a category of deep learning models that specialize in the understanding and processing of sequential data, such as time series. RNNs can remember information from earlier stages, which enables them to understand the patterns and relationships in data over time^13,14^. The internal state of this network can be updated by its feedback loop using the current input and past states to facilitate sequential data processing, as illustrated in Fig. 2. A feedback loop line returning to the RNN block demonstrates the concept of recurrent connection. This allows the network to store and utilize the memory of previous inputs and the current data for functions dependent on sequence and time. The node labeled Xn represents the system’s input, which is directed into the processing cell. This component manages the sequential characteristics of the data by integrating the present input with information from previous time steps. The output of the RNN is denoted as Yn, reflecting its interpretation of the current input in the context of past data.

Simple RNNs have been improved by the development of models like Long Short Term Memory (LSTM) or Gated Recurrent Unit (GRU), which modify the architecture of the neural networks to remember important information over a long sequence of time and avoid the rapid forgetting problem^15,16^. Integrating attention mechanisms and RNN , LSTM , and GRU hybrid models with other machine learning and deep learning has enhanced their forecasting accuracy by capturing time-series patterns^17^. LSTM networks are developed to model temporal dependencies directly in the time domain, unlike CNNs or ANNs. Its architecture allows it to learn the dynamic behavior of wave propagation and reflection directly from raw signals. CNNs can miss long-range temporal dependencies because of pooling layers. They are less sensitive to absolute timing^18^. ELM depended heavily on manual feature extraction and signal transformation^19^. This causes information loss during preprocessing. Traditional feedforward ANNs lack internal memory and cannot capture long-range correlations between wave reflections over time^20^.

### Literature review

Artificial intelligence (AI) methods, including Artificial Neural Networks (ANN), Genetic Programming (GP), and Evolutionary Polynomial Regression (EPR), have been extensively implemented in the prediction and modeling of the behavior of pile foundations^21^. Weiping Liu et al.^22^ modeled a convolutional neural network (CNN) model to characterize pile integrity utilizing low-strain reflected wave images. The systematic CNN addresses the issues of general inefficiency and costly manual inspection by improving generalization and robustness. Xiaolin Li et al.^23^ presents an advanced method for identifying pile defects to improve classification and localization accuracy. De- Mi Cui et al.^24^ introduce a machine learning methodology to automate the analysis of LSIT signals. Key features are collected from reflectogram using wavelet decomposition and then input into the Extreme Learning Machine (ELM) classifier for defect detection. Canhui Zhang et al.^25^ present an innovative methodology that applies two back-propagation artificial neural network (ANN) models to automate the assessment of pile integrity using low-strain dynamic testing. During evaluation, ANN models provide acceptable predictions that address the relationship between PIT input data and the conditions of pile integrity. Silvia García^26^ developed recurrence plots (RPs) to facilitate the analysis of LSIT data, which is derived from chaos theory that converts time series data into a visual representation to show structural defects in piles. Characteristic patterns associated with specific defects are identified in this approach, reducing interpretative subjectivity and enhancing the reliability of pile condition assessments. Kun Meng et al.^27^ constructed velocity response curves under low-strain excitation by presenting a semi-analytical model based on elastic wave propagation. This approach utilizes ontology and semantic web rule language (SWRL) for defect identification and integrity evaluation, which addresses the practical framework and precision, and its application in structural assessment. Haiyuan Wang et al.^28^ developed and validated an AI approach utilizing an RNN integrated with a Multi-layer Neural Network (MLNN) for the classification of LSIT signals and analyzing LSIT signals based on value and order relationships among input features, considered an advantage for RNN till it requires more training parameters and longer training time. Natalia Koteleva et al.^29^ discussed the constraints in simple LSIT for cast-in-place piles, where identifying minor defects could be very consuming and highly dependent on human expertise, using an ANN classifier algorithm to enable automatic recognition and precise localization of defects in these piles. This algorithm involves finding the peaks in signals obtained from the impact of a handheld hammer on the top of a pile, with reflected waves captured by an accelerometer and then processed by the Fourier transform. Övünç Öztürk et al.^30^ focus on improving LSIT by automating the interpretation of reflectogram using transfer learning with CNNs . Deep learning-enhanced LSIT can significantly improve defect detection accuracy, reduce reliance on expert judgment, and enhance integrity evaluations on construction sites. Jing Xiao et al.^31^ enhanced LSIT by introducing a Genetic Back-propagation (BP) neural network to improve the classification accuracy of pile quality based on frequency domain vibration signals. By integrating Genetic Algorithms (GA) with a BP neural network, the hybrid model successfully classified pile conditions, as the field data from pile tests were processed into frequency features and increased prediction speed compared to conventional BP networks.

Loseva et al.^32^ proposed a comprehensive study to improve the resolution of the LSIT for identifying minor defects in cast-in-place piles. The Finite element method with numerical simulations was developed by systematically varying the pulse duration at the input. These simulations demonstrated that the resolution of the technique was significantly increased for short input pulses generated by light hammers with hard heads. Based on these findings, field testing should be carried out using hammers of different weights and head materials to collect data with proper engineering judgment. Ding et al.^33^ address a significant challenge in LSIT for large-diameter pipe piles, specifically those with high-frequency interference. A novel analytical solution for vertical vibratory response under low-strain conditions, validated through 3D FEM simulations and experimental model tests, which successfully captured high-frequency phenomena missed by 1D models. In contrast to frequency, the peak strength of high-frequency interference is mainly affected by external testing conditions. Specifically, using a wider impulse can reduce the intensity of this interference, and stiffer surrounding soil, notably lower Poisson’s ratio, causes smaller peak values. High-frequency interference can often be reduced through practical measures, such as using soft hammers to produce wider impulses and placing receiving sensors radially at 90 degrees to the pile at the impact point, thus minimally distorting the signal to increase the reliability of LSIT results. Zheng et al.^34^ describe unintentional hammer eccentricity as a form of high-frequency interference of reduced accuracy and reliability and misinterpretation of the pile integrity results. It should be noted that when performing LSIT according to the axisymmetric loading, eccentric strains result in complicated 3D wave effects that cannot be previously related to the traditional LSIT, which, for all practical purposes, should be axisymmetric loaded. The amplitude of high-frequency interference varies with the receiver’s angles relative to the circumferential angle and hammer offset position, resulting in minimal distortion of the impact at approximately 90 degrees relative to the impact point. This means the receiver can be at the minimum or maximum radial distance, ranging from 0.5R to 0.7R, to minimize high-frequency interference and improve the reliability of LSIT results. Chai et al.^35^ highlighted that while 1D stress wave theory is commonly used to interpret reflections in LSIT of piles, it may not fully capture the complex wave behavior near the pile top. The study of cylindrical waves is conducted through theoretical models of these waves’ behavior and numerical simulations. If the impact pulse has a wavelength at least four times the pile radius, Rayleigh wave effects locally near the top will be reduced, and the behavior of the wave forms below will be more similar to simply 1D plane waves. If the receiver is not placed too close to the center of the pile (within approximately 0.6 × the pile radius from the center), then it is possible to reliably apply 1D theory to interpret reflections that may occur from deeper within the pile. Luo et al.^36^ proposes a pure convolutional architecture for time series analysis by modernizing the traditional temporal convolutional network (TCN), enabling it to achieve consistent state-of-the-art performance in five mainstream time series tasks. Unlike the recent trend favoring Transformer- and MLP-based models, ModernTCN demonstrates that a well-designed convolution structure can not only match but also surpass these alternatives in both accuracy and efficiency for general time series analysis. Liu et al.^37^ addressed a design allows the model to better capture multivariate correlations via self-attention, achieves state-of-the-art performance on a variety of real-world benchmarks, and demonstrates superior generalization and efficiency compared to conventional Transformer-based and linear forecasting methods. Vanberlo et al.^38^ introduced that self-supervised learning usually improves downstream classification and segmentation performance compared to fully supervised approaches—especially when large amounts of unlabeled data are available. Xie et al.^39^ presents a gradient-enhanced physics-informed neural network (gPINN) method designed to solve the wave equation, improving upon standard PINNs by incorporating gradient information and boundary hard constraints into the network training. The results demonstrate that gPINNs achieve higher solution accuracy and robustness than traditional PINNs, especially when data is limited, as validated through examples comparing both approaches.

Despite the simplicity and rapid field execution of LSIT, generating and analyzing test signals (reflectogram) remains a manual and expertise-intensive task. This research addresses this challenge by applying advanced AI techniques to automatically generate a reflectogram response accurate enough to detect the pile toe location.

## Methodology

**Fig. 3:**
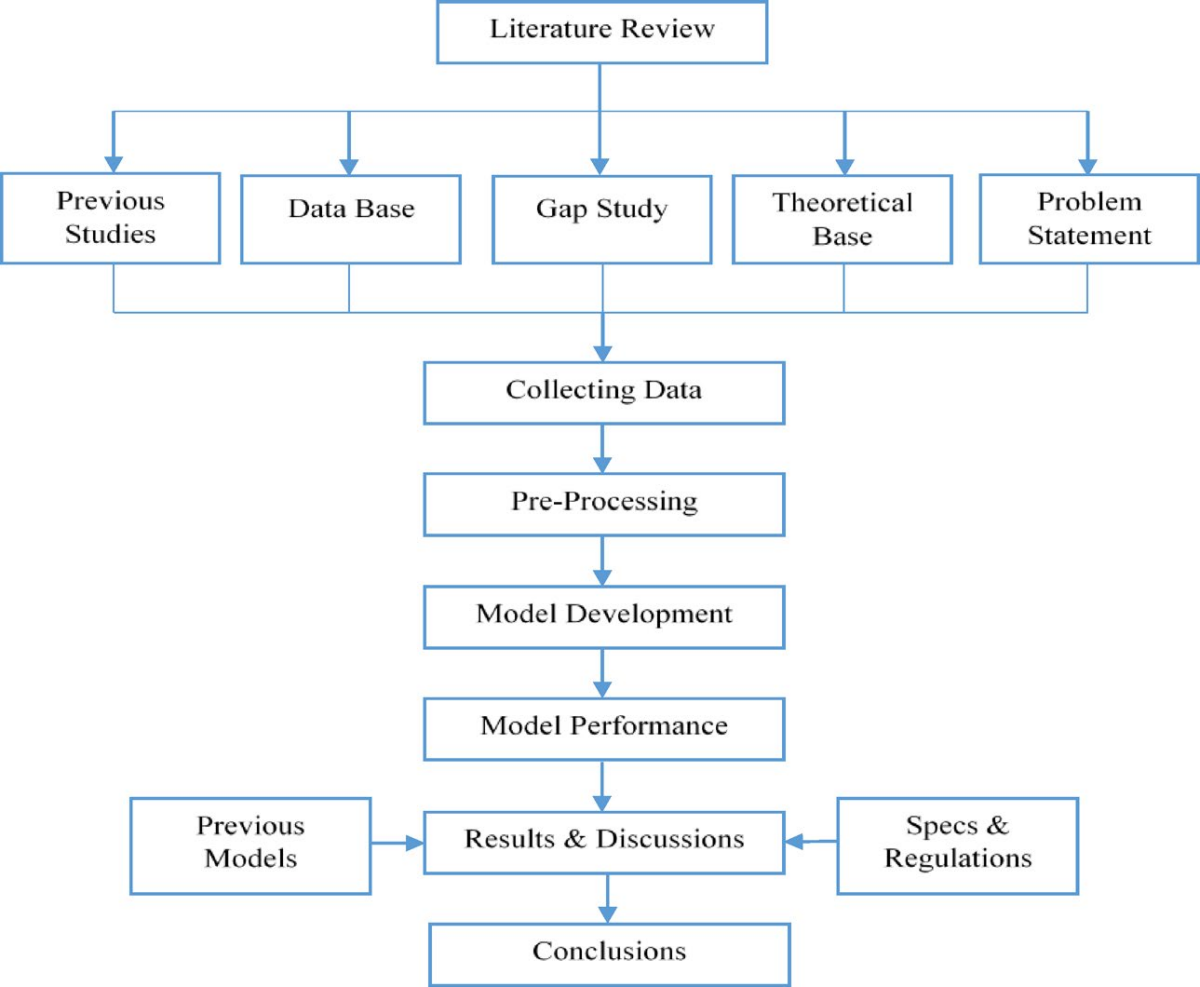
Methodology flow work.

The methodology discussed in this research follows a structured approach to develop an AI-based framework of wave propagation-based NDT, particularly LSIT, as shown in Fig. 3.

### Collecting data

Subsequently, the methodology proceeds to data collection, where LSIT was acquired from different projects in Egypt and provided by “Nile Engineering Consulting Bureau” NECB. This deep foundation project involved construction piles with varying layers of soil at various depths. The database comprised 500 LSIT records of bored concrete piles. The pile lengths varied from 12 to 30 m. The soil profile consists of soft to medium silty clay in the upper layers (0–6 m), transitioning to fine to medium sand at deeper depths (up to 33 m). Table 1 shows that the coefficient of variation for toe depth identification ranges from about 0.55–2.07%. This indicates a reasonably consistent estimation of the toe depths across the pile lengths. Standard Penetration Test (SPT) Values ranged from 4 to 50, indicating variable soil stiffness from soft to dense layers. The results of 500 tests were collected and divided into two subsets, 400 tests for training (75%) and 100 tests for validation (25%) as recommended by^40^. This involved fixed-time-step acceleration signals through the depth of the piles, process reflectogram, and velocity-depth relationships for each tested pile. For machine learning analysis, the collected datasets included input signals that were prepared for training and underwent detailed pre-processing procedures.

**Table 1:** Statistical indicator for consistency of pile toe depth identification.

Pile Length (m)	No. (Pile)	Max (m)	Mean (m)	Min (m)	S.D. (m)	Var (%)
12	29	12.2	12.0	12.0	0.08	0.65
14	23	14.3	14.1	14.0	0.08	0.55
16	63	16.4	16.1	15.0	0.20	1.26
17	62	17.6	17.1	16.6	0.18	1.04
19	47	19.3	19.1	18.8	0.11	0.59
20	22	21.0	20.1	19.4	0.42	2.07
22	35	22.3	22.1	21.8	0.13	0.61
23	28	23.3	23.1	22.9	0.14	0.59
24	42	24.6	24.2	23.9	0.20	0.82
25	95	25.7	25.0	24.6	0.22	0.88
27	26	27.6	27.2	26.9	0.17	0.61
28	8	28.8	28.2	27.8	0.29	1.02
29	12	29.6	29.0	28.7	0.32	1.09
30	8	30.6	30.1	29.6	0.29	0.97

### Data preparation

The collected inputs were text files that include variable length list of raw measured acceleration with fixed time step. While the corresponding outputs were hard copy reflectogram images (unscaled velocity on the vertical axis and depth in meters on the horizontal axis). Both inputs and outputs were preprocessed to be presented in as usable format. First, all reflectogram images were scanned, digitized, and scaled using WebPlotDigitizer^41^. The scanning printed charts’ resolution was 320 × 240 pixels. The data points were automatically extracted using an averaging window with ∆X = 1px and ∆Y = 1px on the reflectogram. The selected reduced time step is equivalent to a length of 0.1m. This is the smallest feature that could be detected, which can be reliably detected and is practically sufficient. The horizontal axis was converted from depth (in meters) to time (in seconds) using the reported wave velocity in the input text files. Since the exact velocity value on the vertical axis has no impact on interpreting the results, the unscaled velocity on the vertical axis was normalized assuming the maximum positive value equals to 100.

**Fig. 4:**
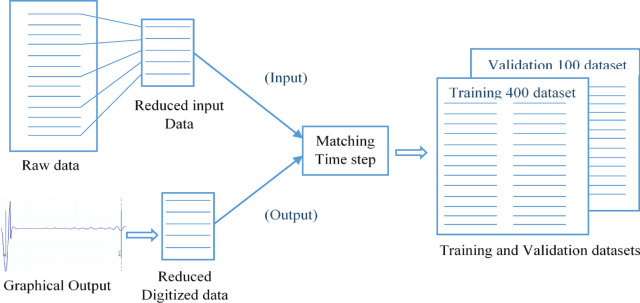
Data preparation procedure.

On the other hand, the input text files included very dense records (more than 1000 records per test). The time step in the input files is very small compared with the time step of the digitized reflectogram images. Hence, the number of records per test from the input text files was reduced to match the number of digitized points from the corresponding reflectogram image (Data reduction and time step matching). Finally, the preprocessed inputs and outputs were combined in one that includes the recorded acceleration and scaled velocity values for each digitized time step. Figure 4 summarizes the Data preparation procedure.

## MODEL development

### Model architecture optimization

**Fig. 5:**
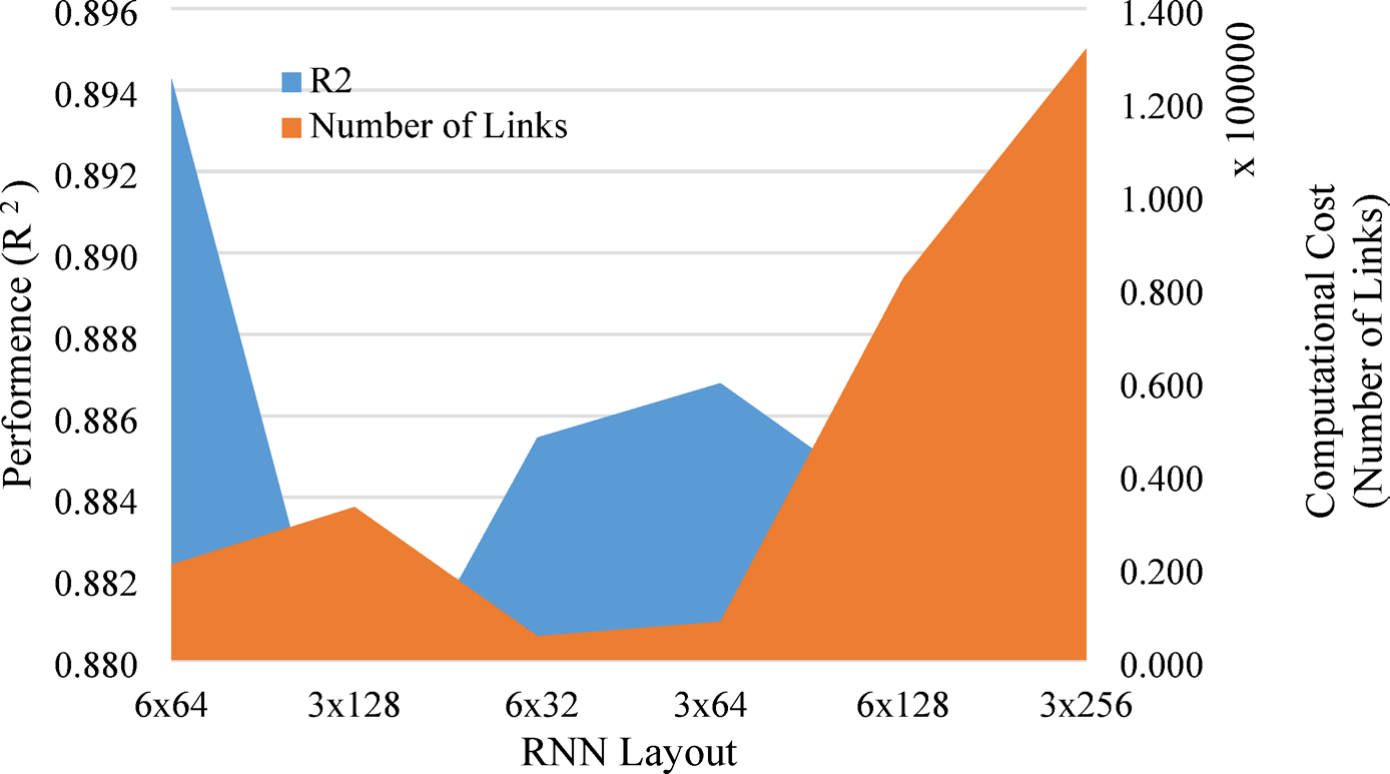
Optimizing the model layout.

In the model development phase, the relationship between acceleration input and velocity output signals from the LSIT data was modeled using an RNN with LSTM (RNN-LSTM) architecture to determine the optimal model configuration for generating integrity pile reflectogram. Architecture design models have been checked with either three or six hidden layers. Each model was performed with different numbers of neurons: 192, 384, and 768. “Anaconda navigator + Jupyter Notebook 7.2.2” package was used to develop all models. These numbers were arranged in layers (Number of layers x Number of nodes) as follows: 6 × 32, 3 × 64, 6 × 64, 3 × 128, 6 × 128, and 3 × 256 nodes. In this context, the numbers 32, 64, and 128 indicate the number of hidden neurons in each LSTM layer, which determines the size of the internal state and how well the model can get time-based information from the input. These configurations were analyzed based on their performance in predicting speed from acceleration signals compared to data from tested piles. Each model was examined using the average value of R^2^ for 75 randomly selected records, the total number of trainable parameters (nodes and links), and a derived performance as shown in Fig. 5.

**Fig. 6:**
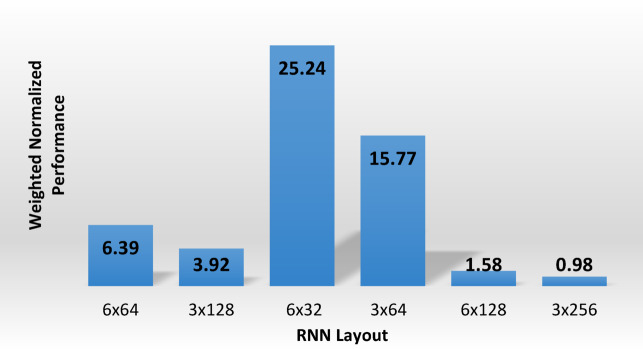
Comparison of model layouts of optimal performance and computational cost.

Among all tested models, the 6-layer, 32-neuron model (6 × 32) had a desirable balance between prediction performance, model complexity, and computational cost with a focus on generalization to unseen data inputs. Wider networks, such as 6 × 64 and 3 × 64, had higher peak R ^2^ values, but the 6 × 32 model regularly had competitive R^2^ values with fewer parameters, as shown in Fig. 6. This makes it less likely to over fit and better for use with fewer computational costs. The final architecture has six LSTM layers with 32 units each. Table 2 summarizes the results of the considered layouts.

**Table 2:** The results of the considered layouts.

Layout (Number of layers x Number of nodes)	6 × 64	3 × 128	6 × 32	3 × 64	6 × 128	3 × 256
Average performance (R^2)^	0.894	0.877	0.885	0.887	0.884	0.876
Normalized performance (R^2^/R^2^ max)	1.00	0.98	0.99	0.99	0.99	0.98
Computational cost (No. of links)	20,544	32,896	5152	8256	82,048	131,328
Normalized computational cost (No. of links/max No. of links)	0.16	0.25	0.04	0.06	0.62	1.00
Weighted normalized performance (Normalized performance/normalized cost)	6.39	3.92	**25.24**	15.77	1.58	0.98

Significant values are in bold.

### Pre-processing

The proposed model handles LSIT.txt files of time and acceleration data during the training. Each text file has been processed by three steps. First, a Savitzky-Golay^42^ filter smooths the signals, reducing high-frequency noise while keeping peak shapes. Then, interquartile range (IQR)^43^ scaling standardizes the input data, transforming each signal by subtracting its median and dividing by the IQR. Also, data augmentation is implemented in training sets by adding minor random variations to the signals with 0.01 and 0.02 standard deviations to improve generalization and robustness. The data can be distorted by adding zeros to make sequences the same length; consequently, a custom reflection-based padding strategy is used. This keeps the data’s patterns consistent, helping the model deal with sequences of different lengths and making it compatible with the input requirements of the LSTM architecture. As a result, it enhances the model’s ability to generalize by maintaining the statistical properties of the input data across batches, particularly when dealing with variable-length sequences.

### Considered model training

**Fig. 7:**
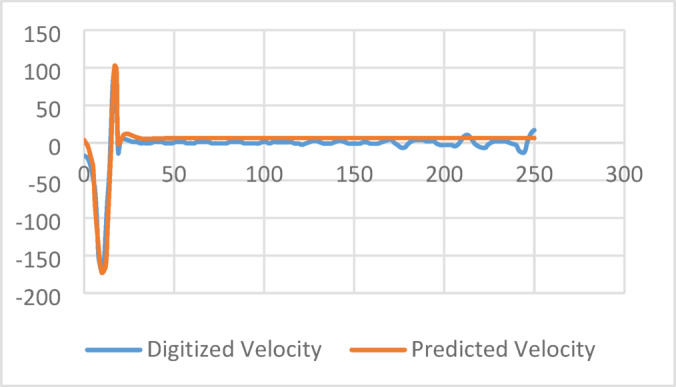
Effect of attention head and residual connection on predictions.

A residual connection joins the second and sixth LSTM outputs. This enhances gradient flow, lowers vanishing gradients, and improves convergence. Additionally, a multi-head attention mechanism (8 heads, key dimension 16) is applied over the residual, followed by a seventh LSTM layer with 64 units. This refines the time-series sequences. Preliminary trials were conducted without these residual connections and attention mechanisms. The model was tested without them and failed to predict the expected digitized velocity reflectogram, as shown in this Fig. 7. As demonstrated in the attached figure, the predicted velocity series (orange) converges to a flat line and does not capture the actual toe reflection event present in the digitized velocity (blue).

**Fig. 8:**
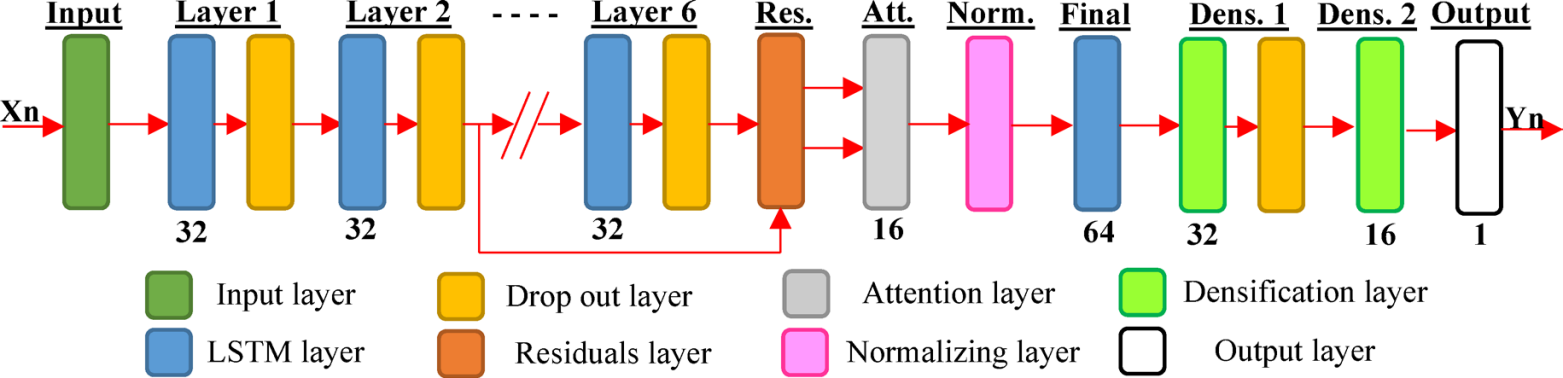
Layout for the considered model.

The output is then passed through two time distributed dense layers with 32 and 16 units, respectively. Rectified Linear Unit (ReLU) activation function is used for each dense layer to introduce non-linearity and enhance the model’s ability to capture complex patterns. An additional dropout layer with a value of 0.1 is applied after the first dense block to prevent over fitting in the final stages. Loss function includes the mean absolute error (MAE) between the predicted and digitized values. MAE provide interpretable evaluation of the network’s performance. This loss function is computed at every epoch during training and validation. This helps guiding optimization and early stopping in the training process. The Adam optimizer is used in training to adjust the learning process efficiently. Using an exponential decay schedule as the learning rate gradually decreases over time to make training more stable and faster. The model was trained for a maximum of 30 epochs, with an early stopping rule used during training as it continuously tracks the model’s performance on the validation set to prevent over fitting. The training stops if the validation loss does not improve by at least 0.0001 over 20 consecutive epochs. Once the training stops, the model’s weights are automatically restored to the weights from that best-performing epoch. This ensures that the final saved model delivers the highest generalization performance based on the training history. Table 3 summarized the considered hyper parameters and Fig. 8 presents the Layout for the considered model, the detailed code is attached in the appendix.

**Table 3:** Hyper parameters for model training.

Dropout after each LSTM layer	0.2
Number of attention heads	8
Dense activation function	ReLU
Optimizer	ADAM
Learning rate	0.001
Max epochs	30
Early stopping patience	20 epochs
Minimum improvement for early stopping	0.0001

### Model performance

The performance of the proposed model is evaluated using:

#### Primary statistical metrics

Statistical metrics such as (MAE, MSE, RMSE & R^2^) were used as primary performance indictors to assess the efficiency of the developed model for both training and validation datasets.

(RMSE) was used to measure the prediction error (the difference between the true and predicted value), while (R^2^) was used to measure the scattering (the difference between the predicted value and the best fitting line). Both metrics are essential to grantee reliable performance assessment.in addition (RMSE) were used to stop the training process before the model becomes over fitted. Equations 3–6 present the mathematical formulas of the used metrics.

3 $${\text{E = }}\frac{{1}}{{\mathrm{N}}}\mathop \sum \limits_{{\text{i = 1}}}^{{\mathrm{N}}} \left| {{\mathrm{y}}_{{\mathrm{i}}} {\text{ - x}}_{{\mathrm{i}}} } \right|$$

4 $${\text{MSE = }}\frac{{1}}{{\mathrm{N}}}\mathop \sum \limits_{{\text{i = 1}}}^{{\mathrm{N}}} \left( {{\mathrm{y}}_{{\mathrm{i}}} {\text{ - x}}_{{\mathrm{i}}} } \right)^{{2}}$$

5 $${\text{RMSE = }}\sqrt {{\mathrm{MSE}}}$$

6 $${\mathrm{R}}^{{2}} { = 1} - \frac{{\sum \left( {{\mathrm{y}}_{{\mathrm{i}}} - {\overline{\mathrm{x}}}} \right)^{{2}} }}{{\sum \left( {{\mathrm{y}}_{{\mathrm{i}}} - {\overline{\mathrm{y}}}} \right)^{{2}} }}$$

#### Visual inspections

In addition to the primary statistical performance metrics (which are used to evaluate the model performance record by record), visual inspection was added to evacuate the whole PIT as one entity by targeting the model objective (determine the pile toe location). It is done by drawing both digitized and predicted reflectogarms in one chart and compare the toe location. The predicted reflectogram is classified as “Good” if the model successfully predicted the toe position with a tolerance of ± 5%, “Fair” if the model misallocated the toe position with a tolerance of ± 10%, or “Bad” if the model did not detect the toe or had a tolerance of more than ± 10%. The results of this inspection are not used in the training process; hence, it is a redundant evaluation indicator.

## Results and discussion

This research uses an RNN with LSTM rather than the traditional multi-step process that begins with extracting the raw data after the experimental tests, signal reduction, selection of a magnification factor, and applying filters to reduce random noise effects. In some cases, the Profile Method, as an analytical modeling, is implemented before finally generating the reflectogram. Also, the increased intensity amplifications are applied to enhance the interpretation of the signals, which is human experience-dependent^6,44,45^.

This study used three performance indicators to assess the effectiveness of the proposed RNN-LSTM model. The model performance was evaluated to determine its ability to generalize the captured relations using the validation dataset. Also, its prediction accuracies were measured for both training and validation datasets.

**Fig. 9:**
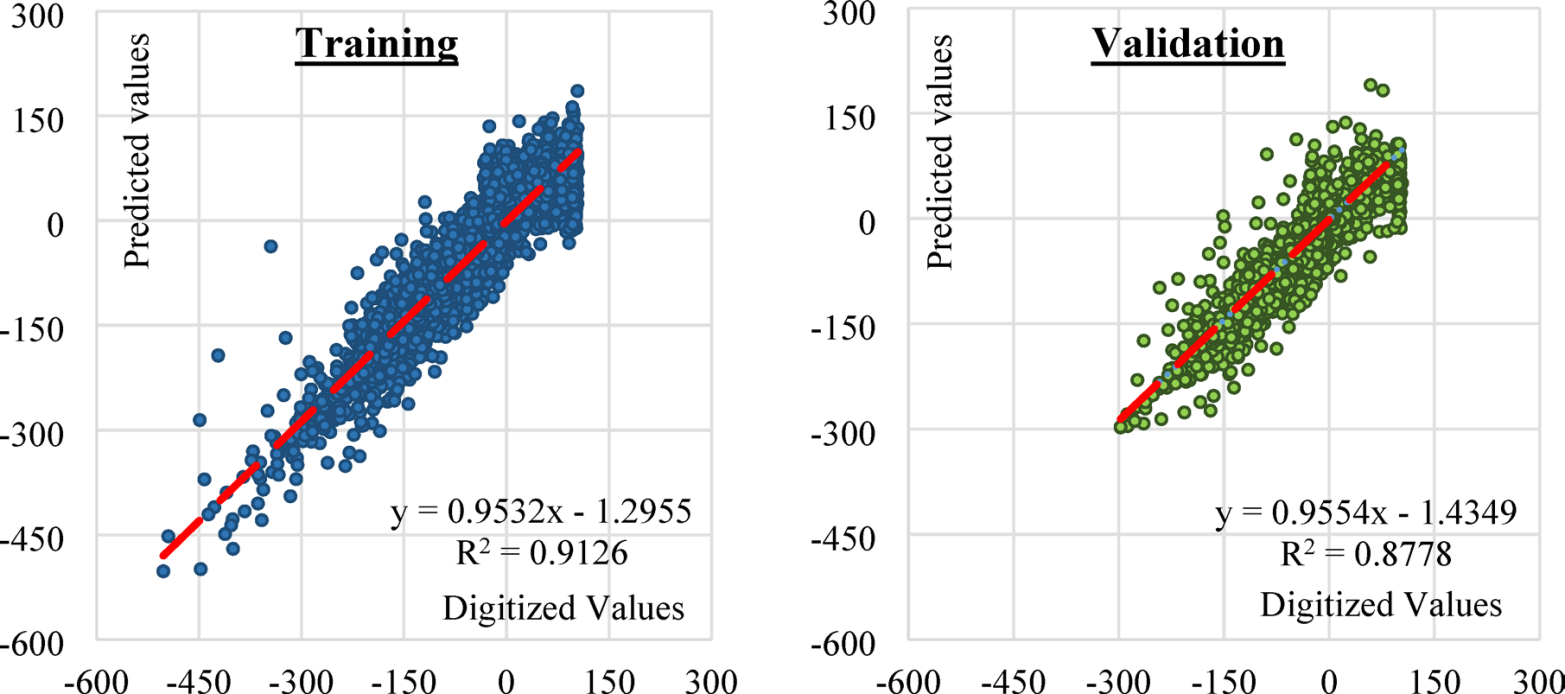
The relationship between predicted and digitized values for training and validation sets.

Figure 9 illustrates the performances of the training and validation datasets as scattering graphs, they showed perfect correlation between predicted and experimental velocity values (R^2^ = 0.913, RMSE = 9.16 for training dataset, R^2^ = 0.878, RMSE = 8.02 for validation dataset). The data points show a strong linear relationship distributed closely around the red best fitting line, with slope of 0.953 for training dataset and 0.955 for validation dataset. The close performances of both training and validation datasets assured the training efficiency. Table 4 summarizes all the performance meters.

**Table 4:** Performance meters.

Meter	Training set	Validation set
MAE	3.75	3.50
MSE	83.90	64.30
RMSE	9.16	8.02
R^2^	0.913	0.878

**Fig. 10:**
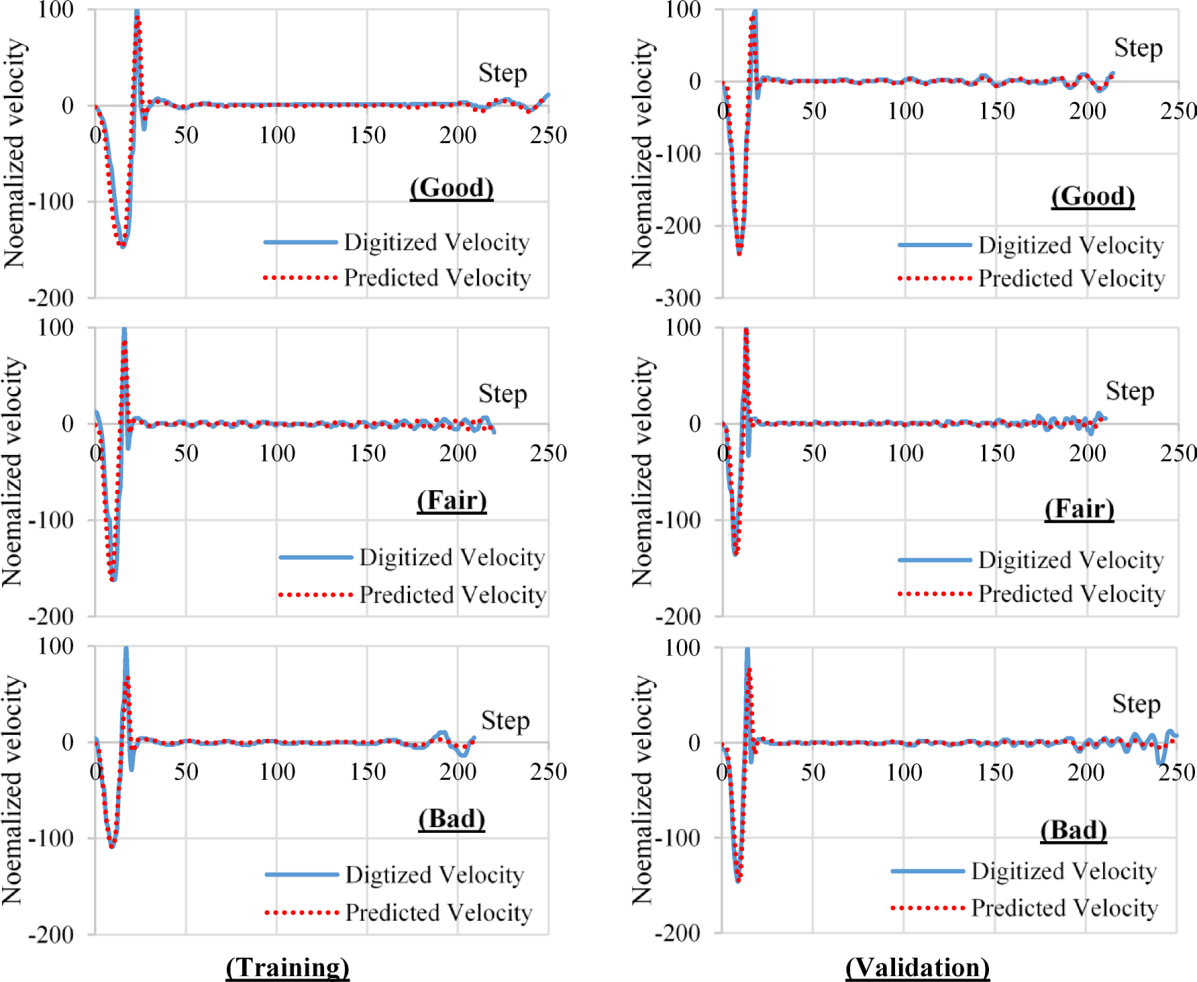
Representative cases of (Good, Fair, & Bad) prediction for visual inspection.

Visual inspection was used to classify the predicted velocity reflectograms into three classes, “Good” if the toe location in the predicted reflectogram matched the location in the digitized one. “Fair” if the toe location in the predicted reflectogram mismatched the location in the digitized one, and “Bad” if the predicted reflectogram did not identify the toe. Figure 10 shows examples for the three classes for both training and validation datasets.

**Fig. 11:**
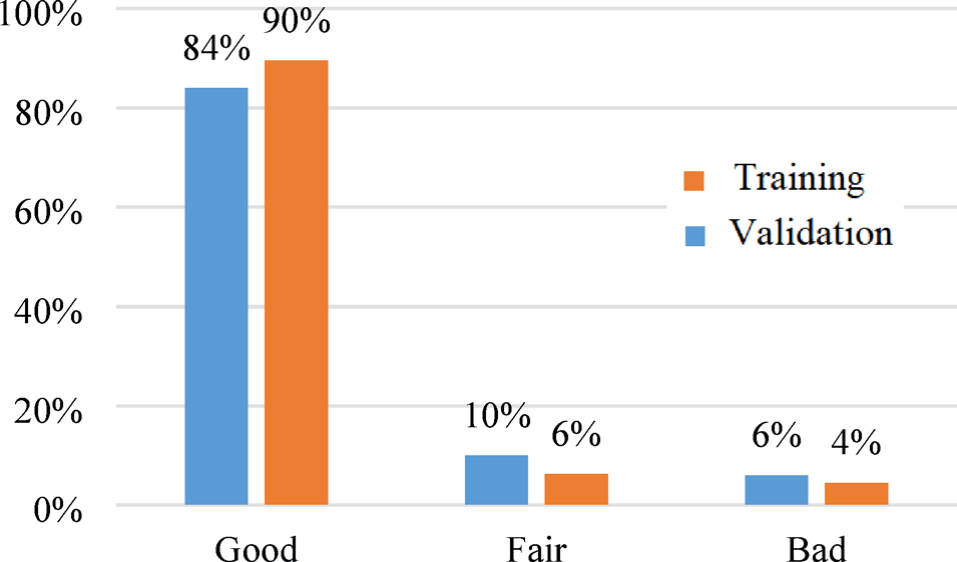
Visual inspection results.

Based on the visual inspection results of the 400 reflectograms of the training dataset were classified into 359 good (90%), 25 fair (6%) and 16 bad (4%). While the 100 reflectograms of the validation dataset were classified into 84 good (84%), 10 fair (10%) and 6 bad (6%). Figure 11 summarized the visual inspection results.

In general, the visual inspection results indicted good agreement between the predicted and the digitized velocity reflectograms for both training and validation datasets.

The commercially available PIT devices are divided into two main types depends on the sensor type. The first type uses accelerometer sensor, while the second one uses velocity sensor (Geophone). This study considered only the first type (with accelerometer sensor) where the outputs are time—acceleration records. Another study may be conducted to consider the second type (with geophone) where the outputs are time-velocity records.

On the other hand, like any ML model, the developed model is reliable within the considered inputs ranges during training process; beyond these ranges, the model must be re-validated to insure its reliability. The considered inputs ranges are listed as follows:

- Pile length 12 to 30 m
- Pile diameter0.8 to 1.2 m
- Concrete strength25 to 40 MPa

Farther studies may be conducted using expanded inputs ranges in order to develop more generalized predictive model.

## Conclusions

This research presents a novel deep learning model to predict the velocity reflectograms of cast in situ concrete piles using pile integrity test raw data. A Recurrent Neural Network with Long Short-Term Memory (RNN-LSTM) was used to replace the traditional multi-steps and human experience based data processing to generate the reflectograms. A primary study was carried out to determine the optimum network layout and hyper parameters, then the main study was conducted using 400 records for training and 100 records for validation. Three performance indicators (RMSE, R^2^ and visual inspection) were used to evaluate the training efficiency and prediction accuracy of the developed model. The proposed technique reduces manual handling, thereby enhancing the accuracy and the performance of the assessment. The study outcomes are concluded as follows:

- The preliminary study showed that the optimum network layout is the one that compromises between performance and computational cost.
- The developed model indicated excellent fitting between predicted and digitized reflectograms with R^2^ of 0.913 and 0.878 for training and validation datasets respectively.
- The model showed good prediction accuracy in terms of toe location (90 and 84% for training and validation datasets respectively)
- The proposed model reduces the dependence on human experience based data processing while ensuring the reliability needed for the LSIT results.

The outcomes of this research are valid only within the considered ranges of inputs (pile diameter 0.8 to 1.2 m), (pile length 12 to 30 m), (concrete strength 25 to 40 MPa). In addition, the developed model was trained using accelerometer readings, hence, it is not valid for geophones readings that provide time–amplitude datasets. As the proposed model is specific to the time-acceleration datasets.

It is recommended for future studies to expand the considered ranges of parameters, develop other ML models to deal with geophones readings and to interpret the generated reflectogram.

## Data Availability

The datasets generated and/or analyzed during the current study are available from the corresponding author upon reasonable request.
